# Can Gender Nouns Influence the Stereotypes of Animals?

**DOI:** 10.3390/ani13162604

**Published:** 2023-08-12

**Authors:** Joao Neves, Inês Costa, Joao Oliveira, Bruno Silva, Joana Maia

**Affiliations:** Department of Science and Education, Zoomarine Algarve, 8201-864 Albufeira, Portugal

**Keywords:** grammatical gender, Stereotype Content Model, emotions, behavioral intentions

## Abstract

**Simple Summary:**

Educating about animals in zoos and aquariums is a challenging task for the education teams. They need not only to be well versed in biology but also have excellent communication skills to convey information effectively to people of different ages and cultures. This study aimed at exploring the possible influence of grammatical genders on animal stereotypes and emotions. For this, four animals (panda bear, giraffe, polar bear, and cheetah) were initially chosen. We then investigated whether the use of grammatical genders in the Portuguese language affected the perceived gender, stereotypes, and evoked emotions of these four animals. To make a comparison, English-speaking participants were also surveyed since English lacks grammatical genders. The results showed that the presence of grammatical genders did influence the perceived gender, as well as the stereotype and elicited emotions of some animals, although the effect was relatively minor. This investigation highlights the importance of small but significant details in communication, such as grammatical genders, in the construction of stereotypes and inherent emotions associated with animals.

**Abstract:**

Educating about animals in zoos and aquariums poses daily challenges for education teams, who must not only master biological content but also possess communication skills to adapt information for diverse ages and cultures. This research consists of two sequential studies designed to investigate the impact of grammatical genders on animal stereotypes and elicited emotions. In Study 1, four animals were independently chosen based on a set of predefined conditions, which were then used in Study 2. The second study explored whether the presence of grammatical genders in the Portuguese language influenced the perceived stereotypes of four animals (panda bear, giraffe, polar bear, and cheetah) using the Stereotype Content Model framework. For comparison, English-speaking participants were also surveyed, as English lacks grammatical genders. The results demonstrated that grammatical genders influenced the perceived gender, as well as, although only slightly, the warmth, competence, and elicited emotions of some animals. All animals under study were associated with the protective stereotype, regardless of the presence of grammatical gender. This study emphasizes the significance of subtle yet crucial elements in communication, such as grammatical genders, in shaping stereotypes and innate emotional associations concerning animals.

## 1. Introduction

Zoos and aquariums are popular destinations that attract a diverse range of visitors, each with their own unique ethnic, cultural, and social backgrounds. These visitors represent a significant portion of the global population, with approximately 700 million individuals visiting zoos and aquariums annually [1]. This presents a valuable opportunity to educate and raise awareness about conservation to approximately 10% of the world’s population [2]. However, educating and raising awareness about animals in zoos comes with numerous challenges for education teams. These challenges extend beyond having a strong grasp of biological content; the teams must also possess effective communication skills to adapt the content for different age groups and cultures, all while ensuring that the visitor’s overall perception remains intact. These challenges can be complex and may involve aspects beyond the content itself relating to the visitors’ comprehension and understanding.

### 1.1. The Weight Words Carry

In addition to interpreting animals and educating about animals, the specific terminology used to name and classify animals can subtly but significantly impact the diverse audiences visiting zoos and aquariums. Words carry cultural associations that reflect and embody the values, beliefs, and norms of the communities that use them. Consequently, words and phrases can possess cultural connotations and associations unique to a particular language, shaping how audiences perceive and interpret concepts and ideas [3]. Moreover, words may have different semantic nuances across languages, with variations in semantic domains and categories leading to differences in the definition and understanding of specific words or concepts. One language may encompass a broader or narrower range of meanings for a word compared to others, influencing the perception and interpretation of those concepts [4]. Furthermore, words can be embedded in idiomatic expressions, where different languages may have distinct expressions and metaphors that convey specific cultural meanings [5]. These expressions contribute to a cultural context that shapes the perception and interpretation of words or phrases, resulting in unique understandings of certain concepts. These examples illustrate how the choice of words can influence the comprehension of important messages depending on the recipient’s language background. It is, nevertheless, important to highlight that while words and language have an impact on perception and understanding, they do not solely determine them. Visitors also bring their personal experiences, beliefs, and cultural backgrounds, which further shape their understanding of words and concepts.

### 1.2. Noun Classes

Grammatical genders, also known as noun classes, are linguistic features that categorize verbs, adjectives, and other elements into fixed categories based on their function within a sentence. It is important to note that the assignment of a grammatical gender, whether masculine, feminine, or neuter, is arbitrary and does not necessarily relate to the actual meaning of the noun or the gender of the entity it represents [6].

In languages of Indo-European origin, there are options for having one (neuter), two (masculine and feminine), or three (masculine, feminine, and neuter) grammatical genders. English, for instance, does not exhibit any distinct gender and is considered neutral in this regard. Languages with two grammatical genders, such as Portuguese, Spanish, or French, classify nouns into masculine and feminine categories. In these languages, each noun is assigned a gender based on its biological or perceived gender, with masculine typically associated with male entities and feminine with female entities. Finally, there are languages that have three noun groups, such as German and Greek, which include options for masculine, feminine, and neuter genders.

From a global perspective, the range of options is diverse. At the far end of the spectrum, languages like Bantu languages (spoken from southern Cameroon eastward to Kenya and southward to the southernmost tip of the African continent) may have more than 10 noun classes. For instance, Swahili can have anywhere from 13 to 18 noun classes depending on the criteria used to define them [7].

### 1.3. Noun Classes and Stereotypes

Noun classes have been observed to influence the perception of social objects in various ways. In languages with two grammatical genders like Portuguese, the gender of a preceding noun can impact the perception of words, potentially attributing certain traits associated with male or female characteristics. For example, the word “teacher” in Portuguese can be preceded by either a masculine or feminine noun, determining whether it refers to a male or female teacher. This gender classification can influence the perceiver to assign gender roles or other stereotypical biases. A recent study conducted by Viana and colleagues [8] found that college students tended to attribute stereotypical traits related to sociability more to female teachers, while perceiving male teachers as more competent and less sociable. However, in the Portuguese language, certain words, such as “chair” or “car,” can also lack a gender counterpart and are exclusively feminine or masculine, respectively. Consequently, they only carry a semantic meaning related to that specific gender.

When it comes to classifying animals, different languages may assign gender, employ specific terminology, or utilize distinct words to describe them. These classifications can carry connotations, associations, or cultural meanings that extend beyond the literal interpretation of the animals themselves. As a result, they can subtly shape attitudes, beliefs, or associations related to the discussed animals.

### 1.4. Stereotype Content Model and Behavioral Tendencies

**Figure 1:**
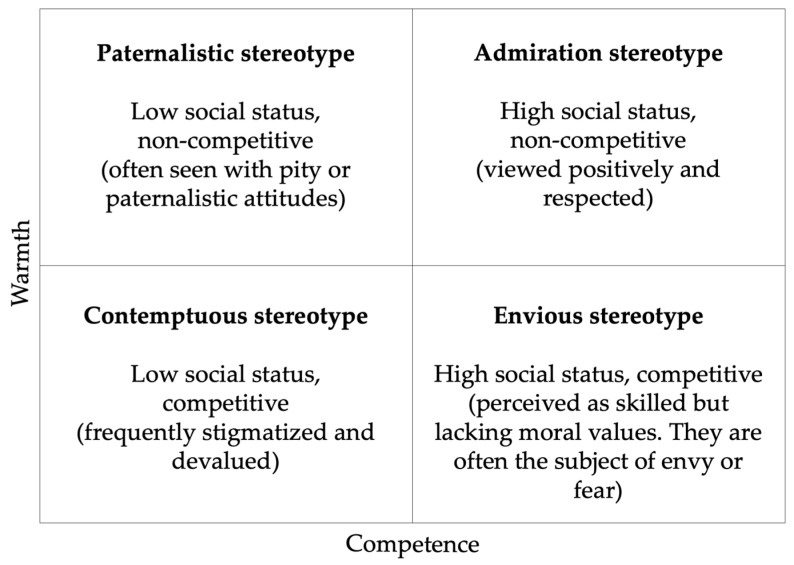
Types of groups, combinations of status and competition, and corresponding forms of prejudice based on warmth and competence (adapted from [9]).

Stereotypes are general beliefs about specific groups of people or other social objects. These beliefs are often based on simplified or incomplete information and can lead to prejudices. The Stereotype Content Model (SCM), initially proposed by Fiske and colleagues [9], provides a framework for understanding how individuals perceive different social groups or objects. According to this model, stereotypes are typically built on two primary dimensions: warmth and competence. Warmth refers to the perception of whether a group or object is friendly, kind, and trustworthy, while competence relates to their perceived skill, intelligence, and capability. Based on these dimensions, groups or objects can be categorized into four distinct stereotypes (Figure 1).

Building upon the SCM, Cuddy and colleagues [10] introduced the Behavior from Intergroup Affect and Stereotype (BIAS) map. This map illustrates how combinations of perceived warmth (low vs. high) and competence (low vs. high) associated with different stereotypes are linked to various emotions (approach vs. avoidance). Ultimately, these emotions lead to intergroup behaviors that can be categorized along dimensions of active vs. passive and facilitative vs. harmful.

### 1.5. Stereotyping Animals

Based on these frameworks, recent studies have demonstrated that animals, similar to other social groups or objects, can be subject to stereotypes (e.g., [11,12,13,14]). In fact, Sevillano and Fiske [11,12,13] have extended the application of the SCM and BIAS map to animals, aiming to describe and understand human–animal relationships. They have identified four predefined stereotypes and associated behavioral intentions for animals: (1) Protective/Companion stereotype (high warmth and competence): This stereotype applies to animals like dogs, cats, horses, and monkeys. It elicits emotions of closeness (e.g., love) and promotes active facilitating behaviors (e.g., interaction, care). (2) Threatening-awe/Predator stereotype (low warmth, high competence): Animals associated with this stereotype, such as tigers, bears, and sharks, evoke a mix of admiration, fear, and avoidance. They can elicit harmful behaviors or active harm (e.g., killing). (3) Subordination/Prey stereotype (high warmth, low competence): Farm animals typically fall into this stereotype, evoking positive emotions (e.g., tranquility) and passive-facilitating behaviors (e.g., ignoring). (4) Contempt/Plague stereotype (low warmth and competence): Invertebrates and similar animals are categorized under this stereotype, generating emotions of avoidance (e.g., disgust) and eliciting active harm behaviors (e.g., killing, poisoning).

Animals can, therefore, carry a set of perceived behavioral intentions that influence people’s predisposition to, for example, support conservation [15,16].

### 1.6. Overview of the Studies

Previous studies have explored children’s perception of gender in animal characters, e.g., [17,18,19]. However, this area of study has not been extensively explored. Moreover, to the best of our knowledge, no study has specifically focused on the potential impact of gendered nouns on the perception of animal gender and their potential influence on stereotypes and elicited emotions among a sample of adult zoo visitors.

Study 1 primarily aimed to choose four animals, out of a pre-selected group of eight, to be included in Study 2. Participants were asked to rate the animals based on their personal preference and were also asked if they knew whether any of these animals were at risk of extinction. Once the four animals were selected, Study 2 focused on exploring whether the presence of grammatical genders in animals could affect how people perceive their gender and stereotype them based on the SCM framework. To investigate this, a group of zoo-goers participated and rated the warmth, competence, elicited emotions, and gender perceptions of these four animals. Two of the animals belonged to the masculine noun class in the Portuguese language, while the other two belonged to the feminine noun class. To make a comparison, English-speaking participants were also asked to complete the same questionnaire. It is worth noting that the English language does not have grammatical genders. The selection of these two languages was consistent with the top two audiences of the park.

In general, our primary objective was to investigate whether the presence of gender nouns, potentially conveying gender-related stereotypes or associations, could influence people’s perceptions of these animals.

The present research adhered to the ethical standards for research involving human subjects as required by the hosting institution. Participants on both studies were provided with information about their rights and the option to discontinue participation at any time without any negative consequences. Prior to participation, all participants signed an informed consent form, confirming their voluntary and anonymous involvement. They were also assured that their confidentiality and anonymity would be respected, in accordance with the ethical principles of the American Psychological Association (APA) and the Portuguese regulations for data protection.

**Study 1**

The objective of this study was to choose four animals, independently and externally, to be included in Study 2. To make the selection, we aimed to address the following straightforward questions: (1) Among the set of eight animals, which ones are most preferred by a general adult audience? (2) Are the preferred animals linked to any specific conservation concern?

## 2. Materials and Methods

### 2.1. Animal Selection

A total of eight animals were chosen for this study based on specific criteria (Table 1). These animals were well-known mammals from medium to large size and were classified in at least one threatened category by the International Union for Conservation (IUCN). None of the selected animals could also have a clearly visible sexual dimorphism aside from the difference in size between genders. In addition, they had a significant presence in popular culture, either through animated characters in movies (e.g., giraffe) or being inherently charismatic (e.g., panda bear). Their dietary preferences were also taken into account, ensuring a balance between carnivores and herbivores. None of the selected animals were present in the collection of Zoomarine Algarve, where Study 2 was conducted. Lastly, from a linguistic perspective, four animals were associated with male grammatical gender nouns in the Portuguese language, while the remaining four animals were associated with female grammatical gender nouns (Table 1 for further details).

### 2.2. Participants and Procedure

A total of 145 Portuguese adults participated in this study, with an average age of 36.64 (*SD* = 13.77). The sample consisted of 92 male participants, with an average age of 35.67 (*SD* = 14.06), and 53 female participants, with an average age of 38.32 (*SD* = 13.24). Study 1 involved an email snowball data collection approach, where participants were asked to complete a short questionnaire. The sample initially included six seeders and expanded through exponential non-discriminative sampling.

First, respondents were asked to rank the eight animals (koala, giraffe, sloth, polar bear, panda, cheetah, elephant, and zebra) based on their personal preference, using a scale from 1 (least preferred) to 8 (most preferred). Next, participants were instructed to select their two favorite animals from the previous list. This additional task aimed to provide an extra criterion for the animal selection, if necessary. Subsequently, participants were asked to identify any animals they believed were threatened with extinction. This simple task aimed to determine if their responses were influenced by known conservation concerns.

Participation in the study adhered to ethical standards for research involving human subjects as required by the host institution. Participants were informed about their rights to participate and the option to withdraw from the study at any time without facing any negative consequences. By checking the consent box, participants indicated their voluntary and anonymous participation. They were assured that confidentiality and anonymity would be maintained in accordance with ethical principles outlined by the American Psychological Association (APA) and Portuguese regulations for data protection.

### 2.3. Preliminary Analysis and Control Checking

Skewness and kurtosis values were analyzed, and all values were below the threshold recommended by [20] (i.e., 2 and 7, respectively).

### 2.4. Data Analysis

One-way ANOVA with Tukey HSD post-hoc tests were used for comparing scores of animal preference. Descriptive analysis was used to interpret animal preference. No significant effect was found after controlling for covariates gender and age.

## 3. Results

The participants indicated that the panda bear was their most preferred animal, followed by the koala and the elephant. Conversely, the sloth and the zebra were the least preferred animals (Table 1). When asked to choose their first preferred animal, the majority of respondents selected the panda bear (25.5%), followed by the koala (21.4%) and the polar bear (15.2%). Among the second choices, the panda bear remained the most popular (22.8%), while the elephant ranked second (19.3%), and the koala ranked third (17.9%) (Table 1). Table 1 also shows that male perceived animals were selected as the preferred ones.

Out of the total 145 respondents, only 7 individuals did not perceive any of the animals as being threatened with extinction. The remaining 138 participants, which accounted for 95.2% of the sample, expressed the belief that at least one of these animals was at risk of extinction. Conservation concern for each animal is presented in Table 1. Among the selected animals, the cheetah received the highest number of votes as being threatened (130), closely followed by the sloth (123). The koala (96), elephant (89), polar bear (70), and giraffe (69) also garnered significant votes indicating perceived conservation concern. On the other hand, the panda (46) and the zebra (37) received the fewest votes in terms of being threatened with extinction.

The results of a one-way ANOVA (*F* (7, 1152) = 20.836, *p* < 0.001; $\eta_{p}^{2}$ = 0.112) indicated a significant difference in animal preference. Table 2 provides further information on the outcomes of a Tukey HSD post-hoc test, which reveals the specific variations between animals.

A total of 18 participants selected the panda bear as their first choice and the koala as their second choice (Table S1). Conversely, for 15 participants, the order was reversed, with the koala as their first choice and the panda bear as their second choice. The third most common pair consisted of the polar bear as the first choice, followed by the panda bear as the second choice, which was chosen by 9 participants. Together, these top three pairs accounted for 29% of the entire sample (Table S1). Notably, all of these pairs of animals were associated with male grammatical gender nouns in the Portuguese language.

## 4. Discussion

Study 1 did not focus on examining participants’ motivations or attitudes toward the animals in detail. Instead, its main purpose was to independently select the animals to be included in Study 2. Although we have successfully achieved the goal of independently selecting four animals, there are still some interesting side thoughts that can be explored.

Out of the eight charismatic animals chosen, seven were also featured in the study conducted by Albert et al. [21], where the authors identified the 20 most charismatic species. Even though the aim of the current study was not to compare studies, our results did uncover a distinct pattern of preference compared to the study mentioned earlier. The panda bear emerged as the top choice, whereas he ranked sixth overall (third when considering similar animals) in the aforementioned study. The second most preferred animal was the koala, who ranked 19th overall (seventh when considering similar animals) in [21]. In contrast, the cheetah ranked second to last in our study (fourth when filtered), followed by the zebra, who ranked sixth when filtered. It is important to note that our study solely focused on animal preference, which explains the disparity in results since [21] considered various other variables, prompting participants to carefully consider their preferences.

One interesting finding is that our top four favorite animals all have masculine grammatical gender. However, due to the nature of our methodology that only asked for the order of preference, it is challenging to infer whether participants’ choices were influenced by the memory of a character from a movie (as these animals have been featured in recent animated films) or recent documentaries or news. Another noteworthy result relates to the animals’ diet. The preference for these four animals may indicate a potential preference for herbivorous animals, as only the polar bear (in fourth place) is carnivorous. Although the dietary aspect was not intentionally emphasized in the questionnaire during the selection process, this finding aligns with other studies that have shown a preference for non-predatory animals (e.g., [22]).

We also aimed to explore whether participants’ choices might be influenced by perceived conservation concerns. Not surprisingly, the animals classified as endangered received the least number of votes on the preference scale (sloth: second to last and cheetah: third to last). Although the conservation status was not explicitly highlighted in the questionnaire during the selection process, this finding aligns with the study by Colléony et al. [23], where animal preference was also unrelated to conservation status. Lastly, it is worth mentioning that both the cheetah and sloth are grammatically feminine in the participants’ language. Although our methodology does not allow us to draw firm conclusions, this raises the question of whether gender role stereotypes may be at play, as females are often perceived as nurturing, empathetic, and cooperative, characteristics associated with the protective/companion stereotype [11].

In summary, although the preference for these animals was not extensively explored in this study, the results successfully achieved their initial objective of selecting four animals from an independent sample.

**Study 2**

In Study 2, following the selection process conducted in Study 1, our objective was to explore whether participants’ native language could influence the gender perception and perceived stereotypes of four animals. We compared two languages: one with gendered nouns (masculine and feminine—Portuguese) and another without gendered nouns (English). Two overarching research questions guided this study: (1) Does grammatical gender influence the gender perception of animals and their perceived stereotypes (specifically in terms of the warmth and competence dimensions)? (2) Given that stereotypes are known to evoke emotions, does the perceived stereotype of each animal align with the emotions predicted by the SCM and BIAS frameworks?

Data were collected from a sample of zoo visitors, as they are typically interested and motivated to learn about animals (e.g., [24,25,26]). We aimed to test the following research hypotheses:

**H1:**  *The presence of noun classes leads to a gendered perception of animals, whereas the absence of noun classes results in a neutral perception of animals.*

**H2:**  *According to the SCM [11] and the social role theory [27], languages with different uses of gender nouns have distinct stereotypical perceptions of animals.*

**H3:**  *According to the BIAS map, different scores of warmth and competence evoke different patterns of elicited emotions and behavioral intentions.*

## 5. Materials and Methods

### 5.1. Participants and Design

A total of 586 zoo visitors were invited to complete a brief questionnaire, with a response rate log maintained throughout the study, indicating a participation rate of 84%. Out of the total, 48 visitors declined to participate, and 48 responses were excluded mainly due to not meeting the language criteria. The final sample for analysis comprised 490 participants (*M*age = 37.39; *SD* = 11.28). Among them, there were 180 male participants (*M*age = 37.04; *SD* = 10.95) and 262 female participants (*M*age = 37.63; *SD* = 11.53) (for a detailed description, see Table 3). All participants were adults and were randomly selected at the entrance or exit of various attractions within the zoo. None of the animals included in the study were present at the zoo during data collection. The study was conducted at Zoomarine Algarve over a period of 54 days, with an average of 9 surveys collected per day.

Based on the results of Study 1, four animals were chosen to be included in Study 2 (Table S2). The selection criteria for these animals were as follows: animal preference > gender noun (in Portuguese) > diet (herbivore vs carnivore). The final list of animals included the panda bear (male + herbivore), the giraffe (female + herbivore), the polar bear (male + carnivore), and the cheetah (female + carnivore).

For each animal, a questionnaire was designed to measure the perceived warmth, competence, emotions, and femininity. Questionnaires were either in the Portuguese or English language. The questionnaire designed for this study was deliberately kept brief and concise. This decision was made considering the data collection process within a naturalistic setting, where potential participants are limited by time constraints that can impact their short attention spans, potentially affecting their willingness to participate.

To assess the warmth and competence dimensions, participants rated each animal using a 7-point scale (ranging from 1 = nothing to 7 = a lot) based on specific traits associated with warmth (e.g., friendly, well-intentioned, warm) and competence (e.g., competent, skillful, intelligent). These items followed the scale used in the SCM developed by Fiske et al. [9] and afterward applied to animals [11].

Emotions (admiration, threat, indifference) were measured by asking participants to rate their feelings on a 7-point scale (ranging from 1 = nothing to 7 = a lot) when seeing or thinking about the selected animal. These emotions were included because they are linked to behavioral tendencies toward animals [11] and have been found to influence conservation intentions, specifically active or passive facilitation, as demonstrated by Neves et al. [15]. The emotion of contempt was deliberately excluded as it does not generate behavioral tendencies toward conservation but rather implies active or passive harm, according to Cuddy et al. [10].

Gender perception was assessed by requesting visitors to rate the animals’ masculinity and femininity on a scale from 0 to 100%. The total of both values needed to equal 100%.

In addition, participants were asked to provide their gender, age, level of education, and mother tongue. One animal was sampled per day, and the daily distribution was randomized. Throughout the study, no photographs of the animals were shown.

The mother tongue information was later filtered according to noun groups for further analysis. The first group of participants consisted of native Portuguese-speaking visitors, while the second group comprised native English-speaking visitors.

### 5.2. Preliminary Analysis and Control Checking

Normality assumptions were met by analyzing skewness and kurtosis values. All values were below the threshold recommended by [20] (i.e., 2 and 7, respectively). All variables displayed good internal reliability (>0.60).

### 5.3. Data Analysis

A one-sample t-test between the midpoint of the scale (3.5) for warmth, competence, emotions, and gender perception (midpoint = 50) was performed. One-sample t-tests were used for comparing both groups in all variables. One-way ANOVA with Bonferroni post-hoc tests were used for comparing multiple samples. Pearson correlation coefficient was computed to assess the linear relationship between variables. GLM analysis was performed for the covariates gender, age, and education for each group. No significant effect was found after controlling for covariates. A 3-way ANOVA was performed to test for the effects of age on the variables of language, sex, and animal. No significant differences were found.

## 6. Results

### 6.1. Gender Perception

In Table 4, the average scores for gender perception by groups are presented for each animal. As anticipated, Portuguese-speaking visitors perceived both the panda and polar bear as males, while the giraffe and cheetah were perceived as females. Surprisingly, English-speaking visitors had a perception that differed from our initial expectations, viewing the giraffe as female and the polar bear as male. The panda and cheetah were perceived as gender-neutral, and these perceptions significantly differed from those of native Portuguese speakers (panda: *t* (113) = −2.61, *p* = 0.01, *d* = 0.540; cheetah: *t* (121) = 2.97, *p* = 0.004, *d* = 0.538).

### 6.2. Perceived Warmth and Competence

**Figure 2:**
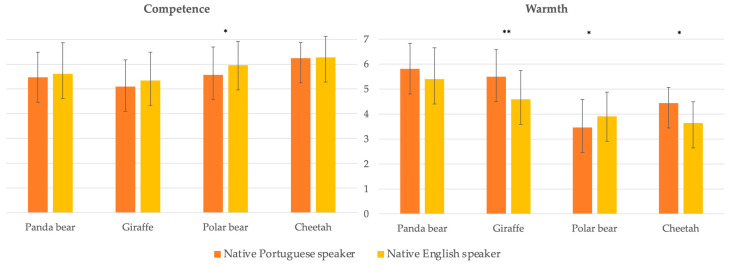
Study’s participants average scores for perceived warmth and competence of each noun group and for each animal. (* *p* < 0.05; ** *p* < 0.001 indicates significant differences between groups). All average scores differed from the midpoint of the scale (3.5) except the polar bear for Portuguese speakers and the cheetah for English speakers.

Panda bear: There were no significant differences between languages in terms of the perceived warmth and competence dimensions (Figure 2). Both stereotype dimensions received high average scores, significantly above the midpoint of the scale.

Giraffe: The giraffe was perceived as highly competent in both languages, with scores well above the midpoint of the scale (Figure 2). However, there were significant differences in warmth between languages (*t* (109) = 4.48, *p* < 0.001, *d* = 0.866), with noticeably higher scores reported in the Portuguese language. Nevertheless, both language groups rated warmth significantly above the midpoint of the scale.

Polar bear: Although both languages rated the polar bear as highly competent, there were differences between them (*t* (133) = −2.16, *p* = 0.032, *d* = 0.371), with English speakers reporting higher values (Figure 2). Warmth displayed a similar pattern, with Portuguese speakers scoring significantly lower than English speakers (*t* (134) = −1.98, *p* = 0.049, *d* = 0.341). Portuguese-speaking participants rated the perceived warmth of the polar bear around the midpoint of the scale, while English-speaking participants rated him significantly above this threshold.

Cheetah: Participants from both groups perceived the cheetah as a competent animal, with similarly high scores well above the midpoint of the scale (Figure 2). However, Portuguese speakers scored warmth significantly higher than English speakers (*t* (119) = 2.99, *p* = 0.003, *d* = 0.556), and both groups rated warmth significantly above the midpoint of the scale.

Despite these observed differences, according to the SCM framework, both groups perceived all animals as belonging to the Protection/Companion stereotype, characterized by high warmth and high competence.

### 6.3. Elicited Emotions

Table 5 presents the average scores of the emotions (admiration, threat, and indifference) reported by participants when thinking about each of these animals.

In general, participants expressed high levels of admiration for all animals surveyed, with no significant differences observed when comparing groups. Similarly, there were no differences between groups in terms of the emotion of threat for three out of the four animals (panda, giraffe, and polar bear). Only the cheetah evoked more threat in native Portuguese speakers compared to native English speakers (*t* (119) = 2.30, *p* = 0.023, *d* = 0.336). Nonetheless, both groups exhibited a similar pattern in terms of average scores for this emotion. Specifically, the panda bear and giraffe received low threat scores, significantly below the midpoint of the scale. On the other hand, the polar bear and cheetah received high threat scores, significantly above the midpoint of the scale.

Indifference received low scores for all animals in both groups, indicating that participants expressed some level of awareness and interest toward all animals. However, the panda bear (*t* (109) = −3.30, *p* = 0.001, *d* = 0.639) and the polar bear (*t* (124) = −2.43, *p* = 0.017, *d* = 0.436) differed significantly between groups, albeit with low scores.

Interestingly, despite the aforementioned small differences, there was a general similar pattern in the elicited emotions for all animals within each group, characterized by high levels of admiration and low levels of indifference. However, there was a notable difference in the perception of threat. Overall, the carnivores (polar bear and cheetah) were consistently regarded as significantly more threatening compared to the herbivores (giraffe and panda bear) (Portuguese speakers: *F* (3, 235) = 33.43, *p* < 0.001, $\eta_{p}^{2}$ = 0.299; English speakers: *F* (3, 225) = 29.22, *p* < 0.001, $\eta_{p}^{2}$ = 0.280).

### 6.4. Correlation between Variables

In native Portuguese-speaking participants, we observed mild to moderate positive correlations between the reported competence of the animals and their admiration scores (Tables S3–S6). However, this pattern was only present in English-speaking participants for the polar bear and cheetah. Interestingly, in Portuguese speakers, there were moderate correlations between warmth and competence scores for all animals except the polar bear (Table S5).

Notably, the gender perception scores of these animals did not exhibit correlations with any variable, except for a mild negative correlation with threat scores for the panda bear (in Portuguese speakers) and the polar bear (in English speakers), both of which were perceived as male gendered. In addition, indifference scores from both languages showed a mild negative correlation with admiration scores for the polar bear.

## 7. Discussion

The results partially support our initial research hypothesis. We identified a clear perception of gender in the four animals under study when grammatical genders were present, aligning with the genders assigned. However, we did observe gender perception in English in two of the four animals, contrary to our expectation of gender absence in all. This finding highlight that, as expected, the social perception of these animals is influenced not only by grammatical construction but also by other factors. It is not surprising, considering that words and language alone do not completely determine perception and comprehension in specific contexts (e.g., [28]). Personal experiences, beliefs, and cultural background also play a role in shaping the perception and understanding of words and concepts, which helps to explain our results.

Considering this and addressing our second research hypothesis, significant differences in the perceived warmth and competence of certain animals were observed across different languages. The polar bear, perceived as male in both languages, displayed significant differences in the two dimensions being studied. Portuguese speakers perceived him with significantly lower levels of competence and warmth compared to English speakers. This disparity was further supported by the lack of correlation between the two dimensions of the SCM (Table S5). As a result, his average warmth scores positioned him on the boundary between the protection/companion stereotype and the threatening/awe stereotype, which has been associated with other large predators like crocodiles, sharks, and wolves in previous studies (e.g., [11,14]). In contrast, native English participants categorized the polar bear perfectly within the protection/companion stereotype quadrant, similar to other large animals, sometimes predators, that are not perceived as a direct threat to humans, such as dolphins (e.g., [15]). Furthermore, the giraffe, perceived as female in both languages, received significantly higher warmth scores from Portuguese speakers. Similarly, the cheetah, gendered as female within Portuguese speakers and neutral to English speakers, also received significantly higher warmth scores, aligning with the social role theory and as initially formulated. These differences suggest the possibility we aimed to investigate: that different languages may stereotype certain animals in distinct ways based on subtle communication details such as gender nouns. Whereas this study does not allow us to determine the complete extent of the influence of gender nouns, it does lay the foundation for more comprehensive research.

Since all animals, regardless of language, conformed to the protective/companion stereotype, we did not find significant differences in the emotions evoked. Instead, a clear pattern emerged, showing high levels of admiration and low levels of indifference and threat for all animals. According to the BIAS map, this protective/companion stereotype is associated with emotional responses of both active facilitation (e.g., helping, protecting) and passive facilitation (e.g., cooperating with, associating with) behaviors. Previous research has linked this set of behavioral intentions with pro-environmental behaviors and concerns for conservation (e.g., [25,29,30]).

Upon closer examination of each emotional response, our research findings confirm that all animals elicited feelings of admiration, consistently obtaining high values across languages. This logical alignment can be attributed to the perception of these animals as charismatic, which evokes positive emotions. Previous studies, like that of Albert et al. [21], have also supported this alignment by attributing positive characteristics such as beauty, impressiveness, and cuteness, all of which received equally high ratings. The emotion of threat arises when we perceive a social entity as competent, powerful, or capable of exerting control or dominance. It evokes feelings of fear, anxiety, or insecurity, resulting in defensive or avoidant reactions. In the context of animals, the competence dimension defined by the SCM relates to the perception of an animal’s capabilities, intelligence, and effectiveness in achieving its objectives. When an animal is perceived as threatening, he often signifies a recognition of his high competence or ability to pose a challenge or harm to humans. This corresponds with the notably high threat scores observed for the two carnivorous animals in our study. Interestingly, only this specific emotion exhibited significant differences between languages, particularly concerning a single predator (cheetah). These findings are consistent with previous research (e.g., [31,32,33]), which associates predators with heightened levels of threat emotions and their influence on attitudes and behavioral intentions regarding conservation. The considerably low threat values reported for the panda and giraffe align with values documented by Albert et al. [21], where these same animals were categorized with almost negligible threat scores. According to Sevillano and Fiske [11], indifference is commonly associated with the subordination stereotype, indicating a lack of strong emotional connection or positive affective response toward the respective animal. Whereas warmth primarily encompasses positive emotions such as admiration and empathy, indifference falls outside of this positive spectrum, signifying a lack of emotional investment or attachment. The significantly low values of indifference (i.e., high significance) observed across all animals, regardless of groups, align with the overall high scores of warmth and competence obtained. In fact, large charismatic mammals are often perceived as appealing and captivating, which can result in heightened positive emotions and reduced indifference compared to less charismatic species [34,35]. Although differences were found between languages (panda bear and polar bear), these variations were not substantial enough to imply significant discrepancies in behavioral intentions.

## 8. General Discussion

Our results provide experimental evidence suggesting that grammatical gender is, to some extent, associated with the perception of animals and may influence their associated stereotypes and emotions. Whereas it is difficult to quantify the exact extent of this association given the nature and objective of our research, it appears that gender perception may influence the perceived warmth and competence of certain animals and elicits corresponding emotions. In addition, our findings indicate that, as anticipated, all animals included in this study were associated with a favorable conservation stereotype, namely the protective/companion stereotype, regardless of the presence of grammatical gender. This stereotype is linked to proactive and supportive behavioral intentions, as these animals are perceived as competent, capable of taking action, and exhibiting friendliness, and positive intentions toward others. Such characteristics foster a sense of connection and willingness to cooperate, resulting in supportive behaviors and engagement in conservation efforts. Both languages exhibited a similar pattern of high admiration scores, aligning perfectly with the identified stereotype. There were some minor yet significant variations between languages, but the overall pattern regarding threat was similar, with higher values observed for carnivores (significantly above the average) and lower values (significantly below the average) for herbivorous animals. As expected, being charismatic animals, none of them evoked feelings of indifference.

The findings suggest that small but significant details can impact the communication of animal-based messaging in multicultural spaces like zoos and aquariums. Although our results do not provide definitive evidence, they suggest the existence of subtle yet noteworthy differences in how specific animals are perceived across different languages.

### Limitations and Future Research

Upon reflection of the study design, it would be valuable to delve deeper and obtain more detailed insights into specific emotions and behavioral intentions related to conservation in order to further explore our conclusions. The questionnaire used in this study was intentionally kept short and concise, considering the need for data collection within a naturalistic context where visitor attention span is crucial and the willingness to participate honestly is paramount. However, we acknowledge that in future studies, providing more comprehensive information on emotions would be necessary to ensure a more robust analysis. Particularly, in Study 2, a significant portion of the results did not yield statistically significant outcomes. Nonetheless, these results provided the research team with a diverse range of findings that contributed to drawing conclusions and provided important food for thought. It is important to note that although the studies were conducted with rigor and validation, the limited sample size imposed certain constraints on the results. For instance, we observed that the warmth and competence dimensions of Portuguese speakers for the polar bear were situated on the boundary between two stereotypes. Expanding the sample size could help to resolve this ambiguity.

In future studies, it may be worthwhile to expand the research to include languages that employ three grammatical genders (e.g., German) to investigate potential differences in animal perception. Within this line of inquiry, exploring whether there are stereotype variations between languages with the same grammatical genders but different gender representations (e.g., the cheetah being feminine in Portuguese and masculine in Spanish and French) would be interesting. Lastly, it would be valuable to examine whether the presence or absence of grammatical gender truly influences the perception of a specific message. This could be accomplished by employing a standardized conservation message and evaluating knowledge acquisition or attitudinal changes across different languages.

## 9. Conclusions

Our perception of animals is influenced by various factors, including cultural influences, past experiences, media portrayals, and personal characteristics. These elements collectively shape our emotional responses and attitudes toward animals. In addition, stereotypes and preconceived notions can impact our initial assessments of an animal’s behavior, often leading to emotional reactions that may not align with his true nature. These emotions, if not properly addressed, can potentially hinder our support for conservation efforts, especially when information goes against established cultural norms. It is worth noting that gender stereotypes in animals have not been extensively studied so far. This study, although straightforward in its design, provides new insights into the subtle influence of communication and social psychology.

## Figures and Tables

**Table 1:** Gender noun, diet, and descriptive statistics for all animals under study.

					N (% of Total)		
Animal	IUCN Classification	GENDER NOUN	Diet	Mean (SD)	1st Choice	2nd Choice	Endangered * (% of Total)
*Panda bear* (*Ailuropoda melanoleuca*)	Vulnerable	Male	Herbivore	5.74 (2.01)	37 (25.5)	33 (22.8)	46 (33%)
Koala (*Phascolarctos cinereus*)	Vulnerable	Male	Herbivore	5.12 (2.25)	31 (21.4)	26 (17.9)	96 (70%)
Elephant (*Loxodonta* spp.)	Endangered	Male	Herbivore	4.97 (1.98)	15 (10.3)	28 (19.3)	89 (64%)
Polar Bear (*Ursus maritimus*)	Vulnerable	Male	Carnivore	4.84 (2.12)	22 (15.2)	17 (11.7)	70 (51%)
*Giraffe* (*Giraffa camelopardalis*)	Vulnerable	Female	Herbivore	4.34 (1.96)	10 (6.9)	17 (11.7)	69 (50%)
*Cheetah* (*Acinonyx jubatus*)	Vulnerable	Female	Carnivore	4.06 (2.59)	16 (11)	13 (9)	130 (94%)
Sloth (*Bradypus* spp.)	Vulnerable	Female	Herbivore	3.60 (2.36)	13 (9)	7 (4.8)	123 (89%)
Zebra (*Equus* spp.)	Endangered	Female	Herbivore	3.31 (1.97)	1 (.7)	4 (2.8)	37 (27%)

*—7 participants did not rate any animal as endangered with extinction.

**Table 2:** Results from the Tukey HSD post-hoc test between animal preference.

	Koala (1)	*Giraffe* (2)	Sloth (3)	Polar Bear (4)	*Panda Bear* (5)	*Cheetah* (6)	Elephant (7)	Zebra (8)
2	0.05							
3	>0.001	0.07						
4	0.95	0.52	>0.001					
5	0.22	>0.001	>0.001	0.01				
6	0.001	0.95	0.61	0.05	>0.001			
7	1	0.21	>0.001	1	0.05	0.01		
8	>0.001	0.001	0.95	>0.001	>0.001	0.06	>0.001	

**Table 3:** Demographic information of the research sample.

		Age	Gender		Education		
	*N*	*M* (*SD*)	Male	Female	Basic	High School	University
Panda bear	117	35.24 (9.57)	40%	60%	8%	44%	47%
Giraffe	112	38.56 (13.35)	42%	58%	7%	41%	50%
Polar bear	137	37.14 (10.71)	43%	57%	4%	45%	50%
Cheetah	124	38.70 (11.11)	41%	59%	5%	48%	45%
Total	490	37.39 (11.28)	42%	58%	6%	45%	48%

45 participants did not report their gender; 53 did not report their education.

**Table 4:** Mean (*M*) and standard deviation (*SD*) values for the gender perception of each noun group and for each animal. (* *p* < 0.05; ** *p* < 0.001 indicates differences between the midpoint of the scale. ^#^ *p* < 0.05).

	Native Portuguese	Native English
	*M* (*SD*)	*M* (*SD*)
Panda bear	45.48 * (16.57) ^#^	52.76 (9.44) ^#^
Giraffe	61.72 ** (19.76)	55.68 * (13.58)
Polar bear	43.50 * (18.28)	46.80 * (11.41)
Cheetah	61.86 ** (20.71) ^#^	51.49 (17.63) ^#^

**Table 5:** Mean (*M*) and standard deviation (*SD*) values of the three emotions (admiration, threat, and indifference) of each noun group and for each animal. (* *p* < 0.05; ** *p* < 0.001 indicates differences between the midpoint of the scale. ^#^ *p* < 0.05; ^##^ *p* < 0.001 indicates significant differences between groups).

		Native Portuguese (*M* (*SD*))	Native English (*M* (*SD*))
Admiration	Panda bear	6.16 ** (1.18)	5.84 ** (1.29)
	Giraffe	5.78 ** (1.17)	5.80 ** (1.27)
	Polar bear	5.73 ** (1.39)	5.77 ** (1.45)
	Cheetah	5.72 ** (1.43)	5.72 ** (1.57)
Threat	Panda bear	2.17 ** (1.62)	2.36 * (1.66)
	Giraffe	2.40 ** (1.78)	1.84 ** (1.61)
	Polar bear	4.54 ** (2.23)	4.69 ** (1.98)
	Cheetah	5.04 ** (2.13) ^#^	4.32 * (2.15) ^#^
Indifference	Panda bear	1.52 ** (1.31) ^##^	2.40 ** (1.45) ^##^
	Giraffe	2.02 ** (1.65)	2.07 ** (1.55)
	Polar bear	2.04 ** (1.80) ^#^	2.84 * (1.90) ^#^
	Cheetah	2.28 ** (1.73)	2.33 ** (1.51)

## Data Availability

Data sharing is not applicable to this article due to privacy and ethical restrictions.

## Supplementary Materials

The following supporting information can be downloaded at: https://www.mdpi.com/article/10.3390/ani13162604/s1, Table S1: Study 1’s Cross Tabulation between the 1st and 2nd choices; Table S2: Animal selection criteria for inclusion in Study 2; Table S3: Correlations between variables for native Portuguese speakers (above the diagonal) and native English speakers (below the diagonal) regarding the panda bear; Table S4: Correlations between variables for native Portuguese speakers (above the diagonal) and native English speakers (below the diagonal) regarding the giraffe; Table S5: Correlations between variables for native Portuguese speakers (above the diagonal) and native English speakers (below the diagonal) regarding the polar bear; Table S6: Correlations between variables for native Portuguese speakers (above the diagonal) and native English speakers (below the diagonal) regarding the cheetah.
