# Supplementary material for: Can Gender Nouns Influence the Stereotypes of Animals?
Source: Animals (Basel). 2023 Aug 12;13(16):2604. doi: 10.3390/ani13162604 (PMC10451744; doi:10.3390/ani13162604)
Supplement: Supplementary file 1 [file animals-13-02604-s001.zip › Table S1.pdf]

Table S1: Study 1's Cross Tabulation between the 1st and 2nd choices

| | Koala (1) | Giraffe (2) | Sloth (3) | Polar Bear (4) | Panda bear (5) | Cheetah (6) | Elephant (7) | Zebra (8) |
|---|---|---|---|---|---|---|---|---|
| 1 | | 4 | 3 | 1 | 18 | 2 | 3 | 0 |
| 2 | 0 | | 1 | 1 | 1 | 0 | 6 | 1 |
| 3 | 3 | 0 | | 0 | 3 | 1 | 5 | 1 |
| 4 | 2 | 0 | 0 | | 5 | 7 | 7 | 1 |
| 5 | 15 | 5 | 1 | 9 | | 2 | 5 | 0 |
| 6 | 5 | 1 | 1 | 3 | 3 | | 2 | 1 |
| 7 | 1 | 7 | 1 | 2 | 3 | 1 | | 0 |
| 8 | 0 | 0 | 0 | 1 | 0 | 0 | 0 | |
