# Supplementary material for: Can Gender Nouns Influence the Stereotypes of Animals?
Source: Animals (Basel). 2023 Aug 12;13(16):2604. doi: 10.3390/ani13162604 (PMC10451744; doi:10.3390/ani13162604)
Supplement: Supplementary file 1 [file animals-13-02604-s001.zip › Table S2.pdf]

Table S2: Animal selection criteria for inclusion in Study 2

Animal	Preference	Gender noun	Diet	Selection
Panda bear	1	Male	Herbivore	X
Koala	2	Male	Herbivore	
Elephant	3	Male	Herbivore	
Polar Bear	4	Male	Carnivore	X
Giraffe	5	Female	Herbivore	X
Cheetah	6	Female	Carnivore	X
Sloth	7	Female	Herbivore	
Zebra	8	Female	Herbivore	
