# Supplementary material for: Can Gender Nouns Influence the Stereotypes of Animals?
Source: Animals (Basel). 2023 Aug 12;13(16):2604. doi: 10.3390/ani13162604 (PMC10451744; doi:10.3390/ani13162604)
Supplement: Supplementary file 1 [file animals-13-02604-s001.zip › Table S3.pdf]

Table S3: Correlations between variables for native Portuguese speakers (above the diagonal) and native English speakers (below the diagonal) regarding the panda bear (* p < .05; ** p < .01)

	1	2	3	4	5	6
1.COMPETENCE		.465**	.553**	.016	.235	-.125
2.WARMTH	.510**		.184	-.188	.018	.039
3.ADMIRATION	.195	.359*		-.019	.003	-.157
4.THREAT	-.138	-.301	-.203		.102	-.251*
5.INDIFFERENCE	-.307*	-.359*	-.382*	.484**		.048
6.FEMININITY	-.17	-.121	.038	.163	.115	
