# Supplementary material for: Can Gender Nouns Influence the Stereotypes of Animals?
Source: Animals (Basel). 2023 Aug 12;13(16):2604. doi: 10.3390/ani13162604 (PMC10451744; doi:10.3390/ani13162604)
Supplement: Supplementary file 1 [file animals-13-02604-s001.zip › Table S4.pdf]

Table S4: Correlations between variables for native Portuguese speakers (above the diagonal) and native English speakers (below the diagonal) regarding the giraffe (* p < .05; ** p < .01)

	1	2	3	4	5	6
1.COMPETENCE		.563**	.336**	-.143	-.149	.152
2.WARMTH	.143		.433**	-.223	-.12	.064
3.ADMIRATION	.275	.045		.055	-.143	.067
4.THREAT	-.108	.03	.084		.472**	-.064
5.INDIFFERENCE	-.207	-.044	.02	.321*		.128
6.FEMININITY	-.168	.096	-.058	.101	-.217	
