# Supplementary material for: Can Gender Nouns Influence the Stereotypes of Animals?
Source: Animals (Basel). 2023 Aug 12;13(16):2604. doi: 10.3390/ani13162604 (PMC10451744; doi:10.3390/ani13162604)
Supplement: Supplementary file 1 [file animals-13-02604-s001.zip › Table S5.pdf]

Table S5: Correlations between variables for native Portuguese speakers (above the diagonal) and native English speakers (below the diagonal) regarding the polar bear (* p < .05; ** p < .01)

	1	2	3	4	5	6
1.COMPETENCE		.035	.443**	.02	-.395**	.035
2.WARMTH	.355**		-.013	-.117	.028	.053
3.ADMIRATION	.601**	.275*		.063	-.390**	.141
4.THREAT	-.074	-.131	-.082		.235	-.12
5.INDIFFERENCE	-.014	.295*	-.239*	-.02		.148
6.FEMININITY	-.053	.211	-.067	-.241*	.141	
