# Supplementary material for: Can Gender Nouns Influence the Stereotypes of Animals?
Source: Animals (Basel). 2023 Aug 12;13(16):2604. doi: 10.3390/ani13162604 (PMC10451744; doi:10.3390/ani13162604)
Supplement: Supplementary file 1 [file animals-13-02604-s001.zip › Table S6.pdf]

Table S6: Correlations between variables for native Portuguese speakers (above the diagonal) and native English speakers (below the diagonal) regarding the cheetah (* $p < .05$; ** $p < .01$)

	1	2	3	4	5	6
1.COMPETENCE		.312*	.290*	.096	.023	-.031
2.WARMTH	.202		.17	-.161	.101	-.129
3.ADMIRATION	.522**	.138		.149	-.277	.02
4.THREAT	-.018	-.149	-.081		-.041	-.004
5.INDIFFERENCE	-.138	-.13	-.234	.322**		-.236
6.FEMININITY	.121	.138	.065	.135	.101	
